# Navigating work life after colorectal cancer: insights into work ability and functioning – a Danish follow-up study

**DOI:** 10.2340/1651-226X.2025.44626

**Published:** 2025-11-30

**Authors:** Pernille Pedersen, Laura S. Berntsen, Annette B. Bräuner, Peter Christensen, Katrine J. Emmertsen, Nina A. Frederiksen, Ismail Gögenur, Marianne Krogsgaard, Michael B. Lauritzen, Ole Thorlacius-Ussing, Therese Juul

**Affiliations:** a Department of Public Health, Aarhus University, Aarhus, Denmark; b DEFACTUM, Central Denmark Region, Aarhus, Denmark; c Department of Surgery, Regional Hospital Viborg, Viborg, Denmark; d Danish Cancer Society Centre for Research on Survivorship and Late Adverse Effects after Cancer in the Pelvic Organs, Aarhus and Aalborg University Hospitals, Aarhus and Aalborg, Denmark; e Department of Surgery, Aarhus University Hospital, Aarhus, Denmark; f Department of Clinical Medicine, Aarhus University, Aarhus, Denmark; g Department of Surgery, Regional Hospital Randers, Randers, Denmark; h Department of Surgery, Zealand University Hospital, Koege, Denmark; i Center for Surgical Sciences, Department of Surgery, Zealand University Hospital, Koege, Denmark; j Institute for Clinical Medicine, Copenhagen University, Copenhagen, Denmark; k Department of People and Technology, Roskilde University, Roskilde, Denmark; l Department of Gastrointestinal Surgery, Aalborg University Hospital, Aalborg, Denmark; m Department of Clinical Medicine, Aalborg University, Aalborg, Denmark

**Keywords:** Colorectal neoplasms, return to work, work performance, survivorship, rehabilitation, vocational, long term adverse effects

## Abstract

**Background and purpose:**

Colorectal cancer (CRC) can affect return to work and sustained work participation. While employment rates have been studied, less is known about how survivors manage work demands after returning, despite frequent long-term symptoms. This study investigated work participation and perceived work functioning 12 and 24 months after surgery.

**Patient/material and methods:**

Data stemmed from a Danish late sequelae screening programme including CRC patients aged ≥18 years who were affiliated with the labour market at diagnosis (2021–2024). Participants reported employment status, work role functioning, and work ability. Clinical data were retrieved from a national database. Multivariable logistic regression models, adjusted for cancer type, sex, and age, assessed factors associated with work functioning.

**Results:**

At 12 months (n = 474) and 24 months (n = 257), 76% and 78% were employed. Just over half reported high work role functioning, and the majority reported high work ability at both follow-up points. Bowel-related problems were associated with lower work role functioning (12 months: odds ratio [OR] 0.35, 95% confidence interval [CI] 0.20–0.62; 24 months: OR 0.40, 95% CI 0.18–0.86) and lower work ability (12 months: OR 0.26, 95% CI 0.15–0.46; 24 months: OR 0.20, 95% CI 0.08–0.51). More advanced cancer stage was also linked to lower work ability.

**Interpretation:**

Most survivors return to work within two years; however, persistent bowel-related problems are associated with reduced work functioning. Rehabilitation should address long-term symptoms to support sustained work participation.

## Introduction

Colorectal cancer (CRC) is among the most common cancers worldwide [1]. Advances in screening and treatment have led to earlier diagnosis and improved survival rates [2–4]. While the median age at diagnosis is around 70 years, incidence rates are increasing among younger individuals [5, 6], leading to a growing number of working-age survivors. Recent Nordic registry data have also shown increasing incidence of early-onset CRC and improvements in 5-year survival among both early- and late-onset patient groups [7]. Return to work (RTW) is therefore a key aspect of cancer survivorship [8, 9].

Previous studies have shown that 60%-73% of CRC patients RTW within 2 years of diagnosis [10–13], while others remain on sick leave, receive disability pensions, or transition into early retirement [10]. RTW supports both economic stability and patients’ psychological and social recovery. It is often viewed as a return to a normal identity [14], and it is associated with improved physical and functional well-being, financial security, and quality of life (QoL) [14–16]. From a societal perspective, RTW helps to reduce productivity loss [17].

Most research has focused on RTW rates and associated factors such as cancer stage, treatment, comorbidities, and sociodemographic characteristics [18–21]. Less is known about what happens after RTW. Work functioning, how well individuals manage their work tasks after returning, has been studied primarily in mixed cancer groups. Existing evidence suggests that psychosocial and work-related factors, cognitive symptoms, and time to RTW may influence work functioning [22, 23], but few studies have examined this among CRC patients [24–26].

This is a critical gap, as CRC patients often experience long-term effects such as bowel dysfunction, fatigue, neuropathy, and cognitive challenges [27, 28], potentially hindering sustained work participation [29, 30]. Work functioning reflects the ability to meet job demands given one’s health status [31]. Yet, rehabilitation rarely includes structured work-related support, partly due to limited evidence on how late effects affect functioning across occupations [32]. As a result, support is often experience-based rather than evidence-based.

The aim of this study was to investigate work participation and perceived work functioning among CRC patients at 12- and 24-months post-surgery. The findings aim to inform targeted, evidence-based support for CRC survivors [33].

## Patients/material and methods

### Study design and data source

Since 2018, CRC patients at six Danish surgical departments (Aalborg, Hjoerring, Randers, Viborg, Aarhus, and Koege) have been screened for late sequelae at 3-, 12-, 24- and 36-months post-surgery. The screening tool consists of validated PROMs covering bowel, urinary and sexual dysfunction, and pain [34]. Work-related PROMs were incorporated in May 2022.

The survey was primarily completed electronically via REDCap [35, 36], with paper versions available if needed.

Clinical data from the national clinical database of the Danish Colorectal Cancer Group (DCCG), which includes >95% of Danish patients with first-time CRC [37], were linked using the unique personal identification number.

### Study population

The study was based on a subsample of the late sequelae screening cohort, focusing on employment and work functioning at 12- and 24-months follow-up (12 mFU/24 mFU). Data were extracted from the database in May, 2025. Patients undergoing surgery between May 2021 and May 2024, were eligible, as work-related items were included in their 12 mFU.

CRC patients aged ≥18 years who underwent resectional surgery at the participating centres were invited to the screening programme. Patients with advanced tumours, defined as tumour invasion beyond the mesorectal/mesocolic fascia, histopathological pT4 tumour involving adjacent structures, or peritoneal carcinomatosis, were not invited, nor were patients unable to understand the participant information or complete the surveys [34].

For this study, only patients affiliated with the labour market at diagnosis, defined as being employed, on sick leave, unemployed, or receiving other temporary social benefits, were included. Those receiving disability or retirement pension at diagnosis were excluded, as they were not expected to re-enter the labour force.

Patients who transitioned to old-age or early-age pension between diagnosis and 12 mFU were excluded from the 12 mFU analysis, as retirement is considered a natural, age-related exit from the labour market. Those who retired between 12 mFU and 24 mFU were included in the 12 mFU analysis but excluded at 24 mFU. Patients who did not complete the screening survey or omitted work-related items were excluded from the analyses. Only patients employed at survey completion were eligible to answer work functioning items.

### Work-related patient reported outcome measures

#### Employment status

Employment status at diagnosis was assessed retrospectively via the 3-month follow-up questionnaire. If missing, data were retrieved from later follow-ups.

Employment status at 12 mFU and 24 mFU was assessed prospectively at the corresponding follow-up time points.

Employment status was categorised as ‘employed’ and ‘unemployed’. The ‘employed’ category included individuals in full-time or part-time employment, in flex jobs (a subsidised employment scheme for individuals with permanently reduced work capacity) or receiving a student grant. The ‘unemployed’ category comprised individuals receiving either temporary benefits or a disability pension. Temporary benefits included unemployment, sick leave, or other forms of temporary social support, while the disability pension referred to permanent benefits.

#### Work role functioning questionnaire

Work role functioning was measured using a 10-item short version of the Work Role Functioning Questionnaire (WRFQ), assessing difficulty in meeting work demands due to physical or emotional health problems during the past 4 weeks [38, 39]. The short version is strongly correlated with the full WRFQ [39].

Responses are rated on a five-point Likert scale ranging from 0 ‘difficult all of the time’ to 4 ‘difficult none of the time’. A ‘Does not apply to my job’ option was included to capture job-specific variability; and coded as missing [38]. If two or more items were missing, the total WRFQ score was set to missing. Scores were calculated by averaging the available items and multiplying by 25, yielding a 0–100 scale, with higher scores indicating better work functioning [38, 39].

Although no validated cut-off values exist, scores were categorised into low (< 75), moderate (75–89), and high (≥ 90) based on existing literature [40]. Due to a small number in the ‘moderate category, it was merged with the low group, resulting in a dichotomous variable: low/moderate versus high.

#### Work ability index

Work ability was measured using a single item from the Work Ability Index (WAI) [41], asking patients to rate their current work ability compared to their lifetime best on a 0–10 scale (0 ‘completely unable to work’, 10 ‘work ability at its best’). The item correlates strongly with the full WAI and is a validated and reliable predictor of sick leave and health-related QoL [42, 43].

Based on existing literature, responses were dichotomised: low (0–7) and high (8–10) [44, 45].

#### Impact of bowel dysfunction on work life

The screening included the item ‘Have you experienced that your bowel function/stoma function has affected your working life?’ with ‘No/not relevant’ and seven ‘Yes’ options: ‘Yes, due to poor bowel/stoma function… (1) I am prevented from working, (2) I have had to change my working hours, (3) I have had to reduce my working hours, (4) I have had to change my work tasks, (5) I have had to change jobs, (6) my relationship with my colleagues has deteriorated, (7) my relationship with my manager(s) has deteriorated. This was followed by a free-text item: ‘Please describe briefly, if your bowel function has affected your work life in any other way’. These items were inspired by Garfinkle et al. [30].

### Exposures

Cancer type was categorised as colon or rectal based on clinical records and registered in the REDCap database. Age was grouped into: < 55, 55–62, and > 62 years. Sex was recorded as male/female.

Impact of bowel function on QoL was assessed using a single item from the screening tool: ‘Overall, how much does your bowel function affect your quality of life?’, dichotomized into: ‘not at all/a little’ versus ‘somewhat/very much’. Pathological tumour stage (pUICC) was obtained from the DCCG database and categorised into 0–2 and 3–4.

### Clinical variables

Clinical variables were included to describe the study population. Data on neoadjuvant and adjuvant cancer treatment were obtained from medical records. Neoadjuvant treatment included chemotherapy and/or radiotherapy (short- or long-course before surgery). Adjuvant treatment was defined as chemotherapy after surgery. Stoma status (temporary and permanent) was classified as currently no stoma or stoma, based on self-report at survey completion.

### Statistical analyses

Sociodemographic and clinical characteristics were summarised for the total study population and stratified by cancer type at 12 mFU and 24 mFU. Descriptive statistics were conducted to assess employment status at 12 mFU and 24 mFU, exploring differences between employed and unemployed patients at each point.

Responses to the bowel dysfunction impact item were presented as counts (%). Free-text responses were categorised using text analysis. Two researchers (LSB and TJ) independently analysed the data; disagreements were resolved through discussion.

WRFQ and work ability scores were non-normally distributed with ceiling effects; therefore, both outcomes were dichotomised as previously described. Multiple logistic regression analyses were conducted to examine the associations between each exposure variables (age, sex, cancer type, pUICC stage, and impact of bowel function on quality of life) and the likelihood of high WRFQ scores and high work ability scores. Each exposure was analysed in a separate model. All adjusted analyses included age, sex, and cancer type as covariates to ensure comparability across models and account for potential confounding.

Sensitivity analyses were conducted excluding participants with UICC stage 4. This was done to account for differences in clinical trajectories and treatment courses among patients with advanced-stage cancer, who may experience substantially different functional outcomes compared to those diagnosed with earlier-stage disease.

A *p*-value < 0.05 was considered statistically significant. Stata version MP 18.5 was used as statistical software.

## Results

**Figure 1:**
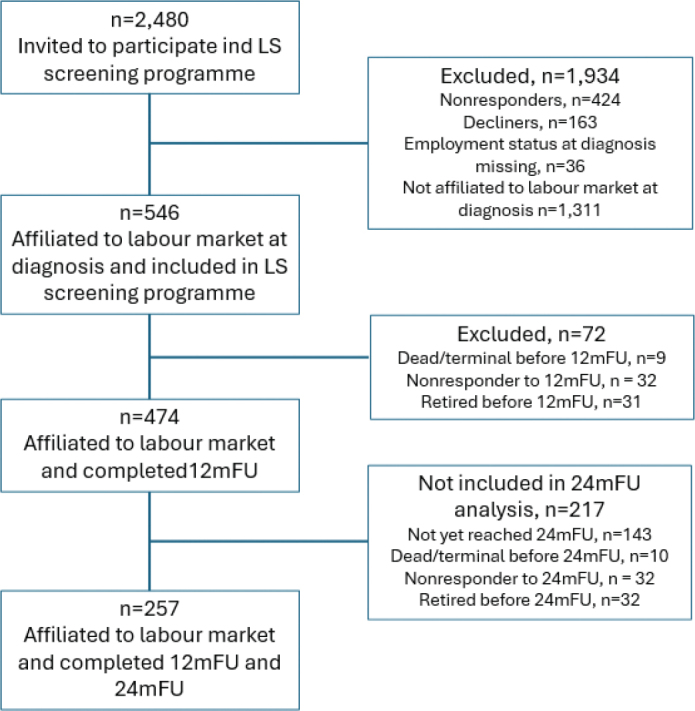
Flowchart of the study.

A total of 2,480 CRC patients underwent surgery and were invited to participate in the late sequelae screening programme (Figure 1). Of these, 1,934 patients were excluded for reasons detailed in Figure 1. The remaining 546 patients were affiliated with the labour market at diagnosis and thus met the inclusion criteria. Among them, 474 remained affiliated with the labour market and completed 12 mFU. Of these, 257 met the same criteria at 24 mFU and were included in the corresponding analyses.

### Characteristics of the CRC patients

Table 1 presents the characteristics of the study population, which at 12 mFU comprised 474 patients, of whom 295 had colon cancer and 179 had rectal cancer. No statistically significant differences were found between 12 mFU and 24 mFU on any variables.

**Table 1:** Patients’ characteristics.

	12 mFU			24 mFU		
	All *n* (%)	Colon *n* (%)	Rectal *n* (%)	All *n* (%)	Colon *n* (%)	Rectal *n* (%)
	474	295 (62.2)	179 (37.8)	257	161 (62.7)	96 (37.3)
**Sex**						
Male	277 (58.4)	163 (55.3)	114 (63.8)	149 (58.0)	92 (58.0)	57 (59.4)
Female	197 (41.6)	132 (44.7)	65 (36.3)	108 (42.0)	69 (42.0)	39 (40.6)
**Age**						
< 55	159 (33.6)	91 (30.8)	68 (38.0)	88 (34.2)	48 (29.8)	40 (41.7)
55–62	184 (38.8)	112 (38.0)	72 (40.2)	109 (42.4)	68 (42.2)	41 (42.7)
> 62	131 (27.6)	92 (31.2)	39 (21.8)	60 (23.6)	45 (28.0)	15 (15.6)
**Neoadjuvant treatment**						
Chemo- and/or radiotherapy	73 (15.4)	21 (7.1)	52 (29.1)	37 (14.4)	9 (5.6)	28 (29.2)
**Adjuvant treatment**						
Chemotherapy	176 (37.1)	126 (42.7)	50 (27.9)	94 (33.6)	69 (42.9)	25 (26.0)
**Stoma**						
Temporary/ permanent	70 (14.8)	12 (4.1)	58 (32.4)	37 (14.4)	7 (4.4)	30 (31.3)
**pUICC stage**						
0/2	264 (55.7)	157 (53.2)	107 (59.8)	146 (56.8)	85 (52.8)	61 (63.5)
3–4	189 (39.9)	123 (41.7)	66 (36.9)	100 (38.9)	67 (41.6)	33 (34.4)
*Missing*	*21 (4.4)*	*15 (5.1)*	*6 (3.3)*	*11 (4.3)*	*9 (5.6)*	*2 (2.1)*
**Impact of bowel function on QoL**						
Not at all/a little	337 (71.1)	235 (79.7)	102 (57.0)	184 (71.6)	126 (78.3)	58 (60.4)
Somewhat/very much	137 (28.9)	60 (20.3)	77 (43.0)	72 (28.0)	34 (21.1)	38 (39.6)
*Missing*				*1 (0.4)*	*1 (0.6)*	

pUICC: pathological tumour stage; QoL: quality of life.

### Employment status

Between diagnosis and 12 mFU, 31 patients retired, and a further 32 retired between 12 mFU and 24 mFU (Figure 1). Among non-retired patients, 76.2% were employed at 12 mFU and 78.6% at 24 mFU (Table 2). Of those unemployed at 12 mFU, 19.8% received temporary benefits and 4.0% were granted a disability pension. At 24 mFU, these figures were 13.6% and 7.5%, respectively. No statistically significant differences in employment status were observed between cancer types at either 12 mFU or 24 mFU (*p* = 0.159 and *p* = 0.627, respectively).

**Table 2:** Characteristics of patients employed at 12 mFU and 24 mFU.

	12 mFU *n* = 474		24 mFU *n* = 257	
	Employed *n* (%)	*p*-value	Employed *n* (%)	*p*-value
*All*	361 (76.2)		202 (78.6)	
**Cancer type**		0.159		0.627
* *Colon	231 (78.3)		125 (77.6)	
* *Rectal	130 (72.6)		77 (80.2)	
**Sex**		0.048*		0.374
* *Male	220 (79.4)		120 (80.5)	
* *Female	141 (71.4)		82 (75.9)	
**Age**		0.910		0.258
* *< 55 years	123 (77.4)		73 (83.0)	
* *55–62 years	139 (75.5)		86 (78.9)	
* *> 62 years	99 (75.6)		71 (71.7)	
**Neoadjuvant treatment**		0.095		0.367
* *Chemo- and/or radiotherapy	50 (68.5)		27 (73.0)	
* *No chemo or radio	331 (77.6)		175 (79.6)	
**Adjuvant treatment**		0.014*		0.013*
* *Chemotherapy	123 (69.9)		66 (70.2)	
* *No chemo	238 (79.9)		136 (83.4)	
**Stoma**		0.002*		0.182
* *Temporary/permanent	43 (61.4)		26 (70.3)	
* *No stoma	318 (78.71)		176 (80.00)	
**pUICC stage**		0.002*		0.001*
* *0/2	213 (80.68)		124 (84.93)	
* *3–4	131 (69.31)		67 (67.00)	
* Missing*	*17 (80.95)*		*11 (100.00)*	
**Employment status at diagnoses**		< 0.001*		< 0.001*
* *Employed	351 (84.2)		192 (84.6)	
* *Unemployed	10 (17.5)		10 (33.3)	
**Employment status at 12 mFU**				< 0.001*
* *Employed	-		189 (94.5)	
* *Unemployed	-		13 (22.8)	
**Impact of bowel function on QoL**		0.005*		0.004*
* *Not at all/a little	274 (81.31)		153 (83,15)	
* *Somewhat/very much	87 (63.50)		48 (66.67)	
* *Missing	0		1 (100.00)	

pUICC: pathological tumour stage; QoL: quality of life.

* Statistically significant difference (*p* < 0.05).

Employment remained generally stable over time, with 65.4% employed at both time points and 18.3% not employed at either. Accordingly, only 16.3% of participants changed employment status between 12 mFU and 24 mFU: 11.8% changed from employed to unemployed, and 4.5% from unemployed to employed (data not shown in table).

Employment at 12 mFU was more prevalent among males, individuals employed at diagnosis, and patients without adjuvant treatment or stoma (Table 2). Similarly, employment was common among patients with less advanced disease (UICC stage I-II) and among those who reported minimal impact of bowel function on QoL (all *p* < 0.048). Similar patterns were found at 24 mFU, although associations with sex and stoma status were no longer statistically significant. In addition, patients employed at 12 mFU were more likely to still be employed at 24 mFU compared to those who were not employed at 12 mFU (*p* < 0.001).

In a sensitivity analysis excluding patients with pUICC stage 4 (*n* = 38 at 12 mFU, *n* = 20 at 24 mFU), associations between employment and sex at 12 mFU, cancer stage at both 12 mFU and 24 mFU, as well as adjuvant treatment at both time points, were no longer statistically significant (all *p* > 0.05).

### Impact of bowel function on work life

At 12 mFU, 19.8% of colon cancer patients and 41.4% of rectal cancer patients responded ‘Yes’ to at least one of the response options 3–9, and/or entered other relevant issues in the free-text (*p* < 0.001) (Table 3). At 24 mFU, the corresponding figures were 19.7% and 36.0%, respectively (*p* < 0.01).

**Table 3:** Frequency of participants reporting impact of bowel function on work life.

		12 mFU			24 mFU		
		All *n* = 457 *n* (%)	Colon *n* = 288 *n* (%)	Rectal *n* = 169 *n* (%)	All *n* = 241 *n* (%)	Colon *n* = 152 *n* (%)	Rectal *n* = 89 *n* (%)
**Have you experienced that your bowel function/stoma function has affected your working life?**							
**1/2**	No, no impact / Not relevant to me	330 (72.2)	231 (80.2)	99 (58.6)	179 (72.5)	122 (80.3)	57 (64.0)
**Responded ‘Yes’ to at least one of the response options 3–9 below, and/or entered other relevant issues in the free text field (option 10)**		127 (27.8)	57 (19.8)	70 (41.4)	62 (25.7)	30 (19.7)	32 (36.0)
**3**	Yes, poor bowel function/stoma function prevents me from working	13 (2.8)	6 (2.1)	7 (4.1)	5 (2.1)	4 (2.6)	1 (1.1)
**4/5**	Yes, I have had to change/reduce my working hours due to poor bowel function/stoma function	37 (8.1)	13 (4.5)	24 (14.2)	15 (6.2)	5 (3.3)	10 (11.2)
**6/7**	Yes, I have had to change my work tasks/job due to poor bowel function/stoma function	27 (5.9)	6 (2.1)	21 (12.4)	10 (4.1)	5 (3.3)	5 (5.6)
**8/9**	Yes, my relationship with my colleagues/manager(s) has deteriorated due to poor bowel function/stoma function	9 (2.0)	3 (1.0)	6 (3.6)	3 (1.2)	2 (1.3)	1 (1.1)
**10**	If your bowel function has affected your work life in any other way, please briefly describe (relevant text entered in free text field)	85 (18.6)	45 (15.52)	46 (27.2)	50 (20.49)	22 (14.5)	17 (19.1)

An analysis of responses to item 10, concerning other ways in which bowel function affected participant’s work life, showed that toilet dependency in the workplace was by far the most frequently reported issue. Other commonly reported challenges included the need for careful planning, such as cancelling travel or avoiding/scheduling meetings to align with bowel emptying routines. Additional issues included embarrassment related to flatulence and borborygmus, as well as fatigue caused by the time and energy required to manage bowel dysfunction.

### Work functioning

**Figure 2:**
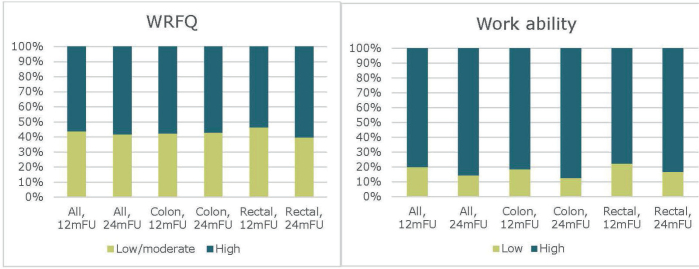
Proportions of patients reporting low/moderate and high scores on the Work Role Functioning Questionnaire (WRFQ) and work ability at 12- and 24-month follow-up (12 mFU and 24 mFU), for the total population and stratified by colon and rectal cancer.

The mean WRFQ score was 73.0 (standard deviation [SD] 35.1) at 12 mFU and 72.2 (SD 36.5) at 24 mFU. Mean work ability was 8.5 (SD 1.6) at 12 mFU and 8.7 (SD 1.3) at 24 mFU (data not shown in table). At both time points, over half of the patients reported high WRFQ score, and approximately 80% reported high work ability (Table 4) (Figure 2).

**Table 4:** Categorised scores on WRFQ and work ability at 12 mFU and 24 mFU.

	12 mFU				24 mFU			
	WRFQ *n* = 312		Work ability *n* = 355		WRFQ *n* = 180		Work ability *n* = 202	
Low/moderate *n* (%)	High *n* (%)	Low *n* (%)	High *n* (%)	Low/moderate *n* (%)	High *n* (%)	Low *n* (%)	High *n* (%)
All	137 (43.9)	175 (56.1)	71 (20.0)	284 (80.0)	75 (41.7)	105 (58.3)	29 (14.4)	173 (85.6)
**Cancer type**								
Colon	85 (42.0)	115 (57.5)	42 (18.6)	184 (81.4)	48 (42.9)	64 (57.1)	16 (12.8)	109 (87.2)
Rectal	52 (46.4)	60 (53.6)	29 (22.5)	100 (77.5)	27 (39.7)	41 (60.3)	13 (16.9)	64 (83.1)
**Sex**								
Male	86 (45.6)	109 (56.4)	39 (18.1)	177 (81.9)	48 (43.6)	62 (56.4)	13 (15.9)	69 (84.1)
Female	51 (44.1)	66 (55.9)	32 (23.0)	107 (77.0)	27 (38.6)	43 (61.4)	16 (13.3)	104 (86.7)
**Age**								
< 55 years	48 (42.9)	64 (57.1)	28 (22.8)	95 (77.2)	27 (43.5)	35 (56.5)	14 (19.2)	59 (80.8)
55–62 years	50 (40.7)	73 (59.3)	25 (18.3)	112 (81.7)	33 (42.3)	45 (57.7)	12 (14.0)	74 (86.1)
> 62 years	39 (50.7)	38 (49.3)	18 (18.9)	77 (81.1)	15 (37.5)	25 (62.5)	3 (7.0)	40 (93.0)
**Impact of bowel function on QoL^a^**								
Not at all/a little	93 (38.4)	149 (61.6)	38 (14.1)	232 (85.9)	51 (37.2)	86 (62.8)	14 (9.2)	139 (90.8)
Somewhat/very much	44 (62.9)	26 (37.1)	33 (38.8)	52 (61.2)	23 (54.8)	19 (45.2)	15 (31.3)	33 (68.7)
**pUICC stage^a^**								
0/2	80 (43.2)	105 (56.8)	30 (14.4)	178 (85.6)	41 (36.6)	71 (63.4)	12 (9.7)	112 (90.3)
3–4	51 (44.7)	63 (55.3)	37 (28.5)	93 (71.5)	28 (48.3)	30 (51.7)	16 (23.9)	51 (76.1)

WRFQ: Work Role Functioning Questionnaire; pUICC: pathological tumour stage; QoL: quality of life.

a Varying *n* due to missing data in ‘Impact of bowel function on QoL’ (24 mFU, *n* = 179) and ‘pUICC stage’ (12 mFU, *n* = 338; 24 mFU, *n* = 191).

Work functioning was stable over time (Figure 2). Among patients with complete data at both time points, 46% had persistently high WRFQ scores and 23% persistently low/moderate scores. Regarding work ability, 76% remained in the high category, while 7% remained low. Most changes over time reflected improvements, though 16% declined in WRFQ score, and 5% declined in work ability (data not shown in table).

Work functioning did not vary significantly by cancer type, sex, or age. However, those reporting that bowel function affected their QoL ‘somewhat’ or ‘very much’ consistently had lower levels of work functioning at both time points. At both 12 mFU and 24 mFU, these patients had significantly lower odds of reporting a high WRFQ score and high work ability, compared to those reporting that function affected their QoL ‘not at all’ or ‘a little’ (Table 5). Patients with a higher UICC stage had significantly lower work ability scores in both crude and adjusted analyses, whereas no significant differences in WRFQ scores were observed across UICC stages. No statistically significant differences were identified across cancer type, sex, or age group.

**Table 5:** Logistic regression analyses of associations between age, sex, cancer type, pUICC stage, and impact of bowel function on quality of life and the likelihood of high work role functioning and high work ability at 12 mFU and 24 mFU.

	12 mFU				24 mFU			
	WRFQ *n* = 312		Work ability *n* = 355		WRFQ *n* = 179		Work ability *n* = 201	
	Crude, OR (CI)	Adjusted^a^, OR (CI)	Crude, OR (CI)	Adjusted^a^, OR (CI)	Crude, OR (CI)	Adjusted^a^, OR (CI)	Crude, OR (CI)	Adjusted^a^, OR (CI)
**Cancer type**								
Colon	ref	ref	ref	ref	ref	ref	ref	ref
Rectum	0.85 (0.54–1.36)	0.83 (0.52–1.33)	0.79 (0.46–1.34)	0.77 (0.45–1.32)	1.12 (0.60–2.06)	1.14 (0.61–2.12)	0.73 (0.33–1.61)	0.79 (0.35–1.77)
**Sex**								
Male	ref	ref	ref	ref	ref	ref	ref	ref
Female	1.02 (0.64–1.62)	0.98 (0.61–1.57)	0.74 (0.44–1.25)	0.74 (0.43–1.26)	1.21 (0.65–2.23)	1.25 (0.67–2.33)	0.82 (0.37–1.82)	0.92 (0.41–2.06)
**Age**								
< 55 years	ref	ref	ref	ref	ref	ref	ref	ref
55–62 years	1.10 (0.65–1.84)	1.08 (0.64–1.82)	1.32 (0.72–2.42)	1.26 (0.68–2.31)	1.05 (0.54–2.06)	1.08 (0.55–2.13)	1.46 (0.63–3.40)	1.42 (0.61–3.34)
> 62 years	0.73 (0.41–1.31)	0.71 (0.39–1.29)	1.26 (0.65–2.45)	1.17 (0.60–2.30)	1.38 (0.60–3.14)	1.46 (0.63–3.38)	3.08 (0.83–11.44)	2.91 (0.77–11.03)
**Impact of bowel function on QoL^b^**								
Not at all/a little	ref	ref	ref	ref	ref	ref	ref	ref
Somewhat/very much	0.37 (0.21–0.64)*	0.35 (0.20–0.62)*	0.26 (0.15–0.45)*	0.26 (0.15–0.46)*	0.49 (0.24–0.99)*	0.40 (0.18–0.86)*	0.22 (0.10–0.50)*	0.20 (0.08–0.51)*
**pUICC stage^b^**								
0/2	ref	ref	ref	ref	ref	ref	ref	ref
3–4	0.94 (0.59–1.51)	0.90 (0.56–1.46)	0.42 (0.25–0.73)*	0.42 (0.24–0.72)*	0.60 (0.32–1.15)	0.62 (0.32–1.19)	0.34 (0.15–0.78)*	0.36 (0.15–0.83)*

WRFQ: Work Role Functioning Questionnaire; pUICC: pathological tumour stage; QoL: quality of life; CI: confidence interval; OR: odds ratio.

a Adjusted for cancer type, sex, and age.

b Varying *n* due to missing data in ‘Impact of bowel function on QoL’ (24 mFU, *n* = 179) and ‘pUICC stage’ (12 mFU, *n* = 338; 24 mFU, *n* = 191).

* Statistically significant difference (*p* < 0.05).

In a sensitivity analysis excluding patients with pUICC stage 4 (*n* = 17/5 for WRFQ and *n* = 18/7 for work ability at 12 mFU/24 mFU), results remained largely unchanged. The only difference was that the crude association between WRFQ score and bowel-related QoL at 24 mFU was no longer statistically significant.

## Discussion and conclusion

In this cohort study based on a late sequelae screening programme among CRC patients, 76% were employed at 12 mFU and 78% at 24 mFU. Employment at 12 months, was more common among males, those employed at diagnosis, and patients who had not received adjuvant treatment or did not have a stoma. It was also associated with lower disease stage and minimal bowel-related impact on QoL. At 24 months, similar patterns were observed, with employment at 12 months being the most consistent predictor.

Overall, work functioning was high: 56% and 58% reported high WRFQ scores at 12 and 24 months, respectively, while 80% and 85% reported high work ability. Bowel-related QoL problems were significantly associated with lower odds of high work functioning at both time points. Higher UICC stage was linked to lower work ability scores, while WRFQ scores did not differ significantly by stage.

We found that 78% of CRC patients were employed at 24 mFU, which is higher than in comparable studies reporting employment rates between 59.7% and 72.8% [10–13]. In a previous study that used national register data including all Danish CRC patients diagnosed between 2001 and 2014, we found that 59.7% were employed 2 years after surgery [10]. That study also showed that patients diagnosed in the later years had higher employment rates, indicating a positive trend over time. This study, which includes more recently diagnosed patients, may reflect a continuation of this development. The higher employment rates observed may relate to earlier detection and improved outcomes following the introduction of national CRC screening in 2014 [46], as well as advancements in treatment and rehabilitation. Methodological differences, particularly patient-reported versus register-based data, and possible selection bias may also contribute to the discrepancy. For instance, a participant working part-time while receiving partial sickness benefits might have reported being employed in the questionnaire, whereas such a person would be categorised as receiving sickness benefits in registry data.

For contextual comparison, the employment rate in the Danish population aged 15–64 years was 76.9% in Q2 2022, according to Statistics Denmark [47]. As our study included participants aged 18 years and older, the true employment rate in a comparable age group may be slightly higher, since younger individuals under 18 are more often in education rather than employment. This suggests that while employment among CRC survivors in our study appears relatively high compared with previous research, it remains close to the level observed in the general working-age population.

To our knowledge, this is the first study to examine WRFQ score among CRC patients. We found mean WRFQ score of 73.0 at 12 mFU and 73.2 at 24 mFU. These scores are lower than in other cancer survivors (85.9) and the general working population (84.2) [23, 38]. Previous studies have shown that late sequelae are highly prevalent among CRC patients [27, 28] with substantial impact on QoL [48, 49]. These difficulties may also impact patients’ ability to meet work demands and likely contribute to the lower WRFQ score observed in our study [30].

Work ability scores were high at both time points (8.5 and 8.7), based on the single-item work ability score from the WAI, which ranges from 0 to 10. This item is not directly comparable to the full WAI, in which total scores range from 7 to 49, our findings are consistent with previous studies. Two studies from the Prospective Dutch ColoRectal Cancer (PLCRC) cohort using the full WAI reported scores of 37.6 and 38 at 24 months post-treatment, respectively, indicating ‘good’ work ability [24, 25]. In contrast, a cross-sectional study also using the full WAI reported a slightly lower score of 34.2, indicating moderate work ability. However, this result may be influenced by the inclusion of unemployed patients in the calculation, which likely contributed to the lower mean scores [26].

Analyses of the responses concerning the impact of bowel dysfunction on work life revealed that 41% and 36% of rectal patients reported that adverse work-related effects of bowel function at 12 mFU and 24 mFU. The corresponding figures for colon cancer patients were around 20% at both time points. These findings are consistent with both clinical experience and previous studies indicating that rectal cancer patients are more severely affected by bowel dysfunction, with significant consequences for QoL [30, 49].

Only patients who were employed at the time of follow-up were included in the analysis of work functioning. While this approach was intended to avoid underestimating work functioning, it may have introduced healthy worker bias. Not working can be considered the most extreme form of poor work functioning [40]. As unemployed patients were excluded from these analyses, the results likely reflect a healthier subgroup of the population, potentially leading to an overestimation of overall work functioning. Furthermore, eligibility for inclusion in this study required resectional surgery, which may have introduced further selection bias by excluding the most frail patients who were not candidates for surgery.

Statistically significant differences were found between colon and rectal cancer patients in several clinical characteristics included in the descriptive analysis. These likely reflect differences treatment strategies, as outlined in national clinical guidelines [50]. Overall, the sample is considered representative of the working-age CRC population, given the relatively high response rate. However, non-responders may differ from participants in important sociodemographic or clinical characteristics, potentially influencing employment status, work role functioning and work ability.

Findings from the sensitivity analyses suggest that patients with pUICC stage 4 constitute a clinically distinct subgroup, particularly with regard to employment outcomes. The attenuation of associations following their exclusion indicates potential heterogeneity between stage 3 and 4 patients. Future research should therefore distinguish between these stages to better capture stage-specific differences in work-related outcomes.

These findings have several implications for clinical practice. Firstly, patients with reduced work ability at 12 months should be identified early and offered targeted support, as recovery appears limited beyond this point. Secondly, when reduced work ability coincides with bowel dysfunction, symptom-specific interventions should be prioritised, given the strong association with impaired work outcomes. Finally, systematic assessment of work functioning, using tools such as the WRFQ, should be integrated into late effects screening or follow-up consultations to address occupational challenges more effectively.

## Strengths and limitations

This study has several strengths. It draws on a clinically relevant cohort from a structured late sequelae screening programme and includes validated, work-related outcomes measures, thereby ensuring high content validity. Furthermore, the use of PROMs contributes important patient-centred insights into the relationship between late effects and work ability.

However, the study also has limitations. The relatively small sample size reduces statistical power, particularly at the 24 mFU, limiting the ability to adjust for all potential confounders. This increases the risk of residual confounding and bias. Moreover, the number of covariates included in the adjusted models may exceed the recommended number of degrees of freedom given the sample size, particularly at 24 months, increasing the risk of overadjustment and model instability. In addition, the inclusion of only employed participants in the work functioning analyses may have introduced a healthy worker bias, potentially leading to an overestimation of work functioning among CRC survivors.

## Conclusion

This study highlights a relatively high level of employment and work functioning among CRC patients up to 2 years after treatment. Employment was associated with sociodemographic and clinical factors, including disease stage, cancer type, and bowel-related quality of life. Notably, patient-reported bowel symptoms were consistently associated with reduced work ability and functioning. These findings underline the importance of addressing late effects in survivorship care and support the integrating of work-related outcomes and symptom burden into routine follow-up, to better facilitate return to and retention in the labour market.

## Data Availability

The data that support the findings of this study are available from the corresponding author, PP, upon reasonable request.
